# Comprehensive Analysis of TSPAN32 Regulatory Networks and Their Role in Immune Cell Biology

**DOI:** 10.3390/biom15010107

**Published:** 2025-01-11

**Authors:** Grazia Scuderi, Katia Mangano, Maria Cristina Petralia, Maria Sofia Basile, Francesco Di Raimondo, Paolo Fagone, Ferdinando Nicoletti

**Affiliations:** 1 Division of Haematology, A.O.U. Policlinico “G. Rodolico—S. Marco”, 95123 Catania, Italy; graziascuderi@hotmail.it (G.S.); francesco.diraimondo@unict.it (F.D.R.); 2 Department of Biomedical and Biotechnological Sciences, University of Catania, 95123 Catania, Italy; kmangano@unict.it (K.M.); ferdinic@unict.it (F.N.); 3 Department of Clinical and Experimental Medicine, University of Messina, 98122 Messina, Italy; mariacristina.petralia@unime.it; 4 Faculty of Medicine, Kore University, 94100 Enna, Italy; mariasofia.basile@unikore.it

**Keywords:** tetraspanins, TSPAN32, immunity

## Abstract

Tetraspanin 32 (TSPAN32), a member of the tetraspanin superfamily, is one of several tumor-suppressing subtransferable fragments located in the imprinted gene domain of chromosome 11p15.5, a critical tumor-suppressor gene region. Although the biology of TSPAN32 remains largely unexplored, accumulating evidence suggests its involvement in hematopoietic functions. In this study, we performed a comprehensive analysis of the expression patterns and regulatory roles of TSPAN32. Notably, TSPAN32 is highly expressed in immune cells, particularly in natural killer (NK) cells and CD8+ T cells. The observed downregulation of TSPAN32 during immune cell activation highlights its potential role as a regulator of immune cell activation and metabolic adaptations, which are crucial for effective immune responses against pathogens and tumors. Moreover, the modulation of biological processes following TSPAN32 knockout further supports its critical role in regulating immune cell physiology and responses. These findings not only shed light on the biology of TSPAN32 but also provide the basis for exploring its diagnostic, prognostic, and therapeutic potential in autoimmune and inflammatory disorders, as well as in hematopoietic cancers.

## 1. Introduction

Tetraspanins, characterized by four transmembrane domains and the ability to form dynamic protein networks known as tetraspanin-enriched microdomains (TEMs), are involved in a wide array of cellular processes, including signal transduction, cell adhesion, and vesicular trafficking [1,2]. Among them, TSPAN32 has recently emerged due to its distinctive expression patterns and regulatory roles in immune cells. Tetraspanin 32 (TSPAN32), encoded by the TSPAN32 gene, located on human chromosome 11p15, is a bona fide member of the tetraspanin superfamily, although it differs structurally from typical tetraspanins by lacking the A/GFLGC motif and having a long C-terminal cytoplasmic domain. However, it retains key tetraspanin features, including polar amino acids and specific cysteine residues in its extracellular domain. The protein also has potential phosphorylation sites, likely with a regulatory role. Alternative splicing of TSPAN32 produces multiple transcript variants, some leading to truncated proteins, possibly targeted for nonsense-mediated decay [3,4].

Studies have highlighted a key role for TSPAN32 in immune regulation [5,6,7,8,9]. In particular, mice lacking TSPAN32 had enhanced T-cell responses, suggesting its ability to negatively regulate peripheral T-lymphocyte proliferation [5,6]. Subsequent studies revealed the involvement of TSPAN32 on different autoimmune disorders, in particular multiple sclerosis (MS) and systemic lupus erythematosus (SLE) [7,8,9]. Indeed, encephalitogenic T cells from Myelin Oligodendrocyte Glycoprotein (MOG)-induced Experimental Autoimmune Encephalomyelitis (EAE) mice—a widely used model for MS—exhibited markedly reduced TSPAN32 expression alongside increased levels of tetraspanins CD9, CD53, CD82, and CD151. Similarly, in MS patients, in vitro-activated circulating CD4 T cells showed significantly lower TSPAN32 levels compared to healthy donors (HD). Moreover, upon antigenic stimulation, myelin-specific memory T cells from MS patients demonstrated significantly reduced TSPAN32 expression compared to those from HD. Peripheral blood mononuclear cells (PBMCs) from drug-naïve MS patients, irrespective of their disease stage, also displayed reduced TSPAN32 levels. Furthermore, a moderate, but statistically significant, decrease in TSPAN32 expression was noted in PBMCs from patients experiencing early relapses compared to those with longer stable disease, suggesting a link between TSPAN32 expression and disease activity [7,8]. In SLE, TSPAN32 expression is significantly downregulated in circulating plasmablasts compared to those from HD. This reduced expression correlates with immune-related gene pathways enriched for type I interferon (IFN)-related genes, highlighting a mechanistic association. Accordingly, IFN-α, a hallmark cytokine in SLE pathogenesis, induces a dose-dependent downregulation of TSPAN32 in B cells. These findings are consistent with observations in IFN-α transgenic mice, where splenocytes also exhibit diminished TSPAN32 expression [9].

In this paper, we provide a comprehensive analysis of expression patterns, regulatory mechanisms, and functional roles for TSPAN32. By elucidating the complex interplay between TSPAN32 and immune cell biology, this study opens new avenues for therapeutic interventions targeting TSPAN32 in immune-related diseases, cancer immunotherapy, and hematological disorders. Ultimately, this work contributes to advancing our knowledge of TSPAN32 biology and its potential therapeutic applications, aiming to improve outcomes in human health and disease.

## 2. Materials and Methods

### 2.1. Expression Levels of TSPAN32 in Normal Tissues

The expression profile of TSPAN32 across normal tissues was obtained from the Genotype-Tissue Expression (GTEx) database (v8, https://gtexportal.org/home/, accessed on 30 May 2024). GTEx provides RNA sequencing data from 54 non-diseased tissue types derived from nearly 1000 individuals, offering robust statistical power with an average of 50–200 biological replicates per tissue type. Expression levels were quantified using Transcripts Per Million (TPM), a normalization method that accounts for both sequencing depth and gene length, ensuring comparability across samples.

Additionally, single-cell RNA sequencing datasets within GTEx, representing over one million cells from multiple tissue types, were analyzed to capture TSPAN32 expression at the cellular level. For single-cell data, log-normalization techniques were employed to scale raw counts, accounting for total read depth and batch effects. A heatmap was generated to visualize TSPAN32 expression across tissues, with hierarchical clustering applied using Pearson correlation as the similarity metric and average linkage as the clustering method.

To investigate the expression of TSPAN32 in different immune cell populations, transcriptomic data from the GSE227743 dataset were analyzed. For the generation of this dataset, different cell types were sorted to high purity using fluorescence-activated cell sorting (FACS). Sorting was performed in two rounds to ensure specificity, isolating approximately 1000 cells per sample. Biological duplicates were prepared for each cell type, yielding two to four replicates per subset. Transcriptomic profiling of these sorted populations was conducted using the ImmGen ULI (ultra-low input) protocol, based on the SmartSeq2 platform. This method was optimized for low RNA input from small cell populations and ensured high sensitivity and reproducibility in gene expression analysis. This dataset encompasses a wide range of sorted immune cell subsets, including immature NK cells (ILC NKimm56hi16-), mature NK cells (ILC NKmat56lo16hi57-), and memory NK cells (ILC NKmem56lo16hi57hi). Among T cells, subsets analyzed included naive CD8+ T cells (T8 NveCD3+8+RA+62L+), activated regulatory T cells (T Tregact), and invariant natural killer T cells (T NKTVa24+). Effector memory T cells, both CD4+ (T4 EffMemCD3+4+RA-62L-) and CD8+ (T8 EffMemCD3+8+RA-62L-), were also included. Monocyte subsets such as Mo16+ and Mo14+ cells were profiled alongside dendritic cell subsets, including DC5AXL + SIGLEC6+, DC1141+, and DC6123+ cells.

### 2.2. TSPAN32 Transcription Factors

Transcription factors regulating the expression of TSPAN32 were identified through a comprehensive analysis of the ENCODE and ChEA ChIP-Seq databases. These databases provide experimentally validated data on transcription factor binding sites, allowing for the identification of factors that influence TSPAN32 expression. Functional and network analysis of the identified transcription factors was conducted using STRING (Search Tool for the Retrieval of Interacting Genes/Proteins), focusing on precomputed local STRING network clusters. STRING clusters are generated through hierarchical clustering of the entire STRING protein–protein interaction network, using an average linkage algorithm to group proteins into functionally related clusters. The smallest clusters contain as few as five proteins, while larger clusters used for enrichment analysis include up to 200 proteins. To avoid redundancy and improve interpretability, clusters with size differences of fewer than five proteins relative to their child clusters (the next smallest in the hierarchy) were excluded. Cluster annotations were automatically assigned based on consensus protein descriptions sourced from multiple functional databases, including Gene Ontology (GO), KEGG pathways, Reactome pathways, UniProt annotations, and structural or domain databases such as Pfam, SMART, and InterPro. Functional enrichment analyses of these clusters were performed to identify biological processes and pathways associated with TSPAN32 regulatory transcription factors.

### 2.3. TSPAN32 upon Immune Cell Activation

To determine the genes correlating with TSPAN32 upon activation of T cells, we used the GSE197067 dataset [10], which included a time-course experiment involving anti-CD3/CD28 bead-activated Pan T-cells from four healthy subjects.

Briefly, for the generation of the GSE197067 dataset, peripheral blood mononuclear cells (PBMCs) were isolated from buffy coats using density gradient centrifugation. Non-T cell populations were depleted using magnetic cell sorting (MACS), and CD3+ T-cells were selected. The purity of isolated T-cells was confirmed by FACS. The cells were activated by adding 2 × 10^5^ Dynabeads Human T-Activator CD3/CD28 per sample. RNA-seq was performed at multiple time points: before activation (0 h) and at 6, 12, 24, 48, and 72 h after activation. As a control, unactivated Pan T-cells were sequenced at the same time points. For RNA sequencing, up to 0.5 × 10^6^ cells per sample were lysed and RNA was extracted using the miRNeasy Mini Kit (Qiagen, Hilden, Germany). RNA quality and concentration were determined with a Bioanalyzer 2100 (Agilent, Santa Clara, CA, USA) and Qubit assay. Libraries were prepared with the TruSeq Stranded Total RNA Sample Prep Kit (Illumina, San Diego, USA) and sequenced using the HiSeq 2500 platform with 2 × 126 bp paired-end reads. Data were processed by demultiplexing the raw sequencing files, trimming low-quality reads, and mapping the reads to the human genome (GRCh38/hg38). Gene expression was quantified using HTSeq, and the data were normalized using EdgeR in counts per million (CPM) for subsequent analysis.

To identify genes correlating with TSPAN32 in NK cells, we used the GSE22886 dataset [11], which contains whole-genome transcriptomic data from NK cells both at baseline (time 0) and at 16 h following treatments with IL2 (10 nM) or IL15 (10 nM). The data were generated using Affymetrix^®^ HGU133A and HGU133B GeneChi)ps (Affymetrix, Santa Clara, CA, USA, which provide robust and high-density gene expression profiling. For our analysis, genes were selected based on a Pearson correlation coefficient of greater than 0.7 or less than −0.7 to capture both positively and negatively correlated genes with TSPAN32 expression. Additionally, we applied a false discovery rate (FDR) threshold of <0.05 to control for multiple testing errors.

Gene set enrichment analysis (GSEA) was performed to identify the biological pathways associated with the genes correlating with TSPAN32 expression. This analysis was conducted using the WebGestalt web-based platform (https://www.webgestalt.org/, accessed on 15 june 2024) [12], which provides an accessible tool for functional enrichment analysis across various biological contexts. For this analysis, we used the Hallmark gene sets from the Molecular Signatures Database (MSigDB), which represent a collection of gene sets that capture key biological states and processes across a broad range of experiments [13]. The Hallmark sets are highly curated to reflect well-established biological themes and provide a more accurate interpretation of gene expression patterns compared to other gene set collections [14]. WebGestalt applies a hypergeometric test to evaluate the statistical significance of the overlap between the gene list and predefined pathways in the Hallmark sets. The results were considered significant if the FDR was below 0.05. The Normalized Enrichment Score (NES) is a critical metric in gene set enrichment analysis (GSEA) that quantifies the enrichment of a gene set within a dataset while adjusting for variations in gene set size and dataset characteristics. It measures the overrepresentation of a predefined set of genes, such as those involved in a specific pathway or biological process, at the extremes of a ranked gene list, which is typically ordered by association with a phenotype (e.g., fold change or statistical significance). To compute the NES, an initial enrichment score (ES) is calculated based on the ranking of genes, followed by normalization to account for gene set size and dataset variability. This normalization is achieved by comparing the observed ES against a null distribution generated through random permutations of the dataset, thereby ensuring that the NES reflects true biological significance rather than statistical artifacts. A positive NES indicates that the gene set is enriched at the top of the ranked list (upregulated), while a negative NES indicates enrichment at the bottom (downregulated), with higher absolute values corresponding to stronger enrichment. The NES provides a standardized and robust measure of enrichment, enabling comparison across gene sets of varying sizes and diverse datasets. The false discovery rate (FDR) is used to assess statistical significance.

### 2.4. Perturbed Biological Process upon TSPAN32 Genetic Ablation

The consensus gene signature for TSPAN32 knockout (KO) was obtained from the Enrichr web-based platform (https://maayanlab.cloud/Enrichr, accessed on 15 June 2024) [15], using the LINCS_L1000_CRISPR_KO_Consensus_Sigs dataset. The LINCS_L1000_CRISPR_KO_Consensus_Sigs dataset is part of the Library of Integrated Cellular Signatures (LINCS) project, which aims to provide insights into the cellular responses to various perturbations, including genetic knockdowns. This specific dataset compiles consensus gene signatures derived from CRISPR knockout experiments across a range of cell types. The dataset includes data on over 4300 gene knockdowns and is used to study the molecular impact of individual gene deletions. The consensus signatures are calculated by averaging the results from multiple replicates of the same perturbation across different cell lines, providing a robust representation of the effects of gene knockouts. To investigate the functional consequences of TSPAN32 knockout, we analyzed the genes that were either upregulated or downregulated in response to its deletion. The upregulated and downregulated genes were subjected to Overrepresentation Analysis (ORA) using the EnrichR web-based platform. This method assesses the enrichment of gene lists in predefined biological categories and pathways. Separate analyses were performed for upregulated and downregulated genes. The Hallmark gene sets from the Molecular Signatures Database (MSigDB) were used for the ORA, with terms considered significantly enriched if the FDR was below 0.05. A combined score was also provided for each enriched biological process in order to integrate both the *p*-value and the z-score of deviation from the expected rank.

To visualize the relationships between the enriched pathways and their associated genes, network construction was performed using Cytoscape software v. 3.10.3 [16].

## 3. Results

### 3.1. TSPAN32 Expression Across the Tissue

**Figure 1:**
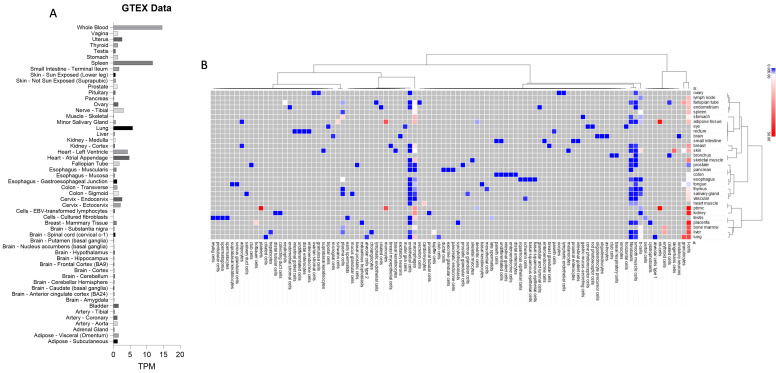
Expression levels of TSPAN32 in normal cells and tissues. Transcriptomic levels of TSPAN32 in normal tissues, as obtained from RNA-Seq data from the GTEX database (**A**); expression levels of TSPAN32 from single cell RNA-Seq data from the GTEX database (**B**).

The expression levels of TSPAN32 indicate predominantly low to no expression across most tissues (Figure 1A). Notably, significant expression is observed in the immune system, particularly in whole blood and the spleen, suggesting a crucial role for TSPAN32 in immune functions. Other organs with moderate expression include the heart, both in the atrial appendage and in the left ventricle. Most other tissues, such as various regions of the brain, liver, and kidney, display much lower expression levels, indicating limited to no expression of TSPAN32 in these organs (Figure 1A).

Single-cell analysis of normal human tissues revealed the expression profile of TSPAN32 across various cell types (Figure 1B). Notably, TSPAN32 exhibits high expression in immune cells, particularly in NK cells (46.1 TPM in adipose tissue, 84.7 TPM in PBMC), monocytes (36.2 TPM in adipose tissue, 37.1 TPM in PBMC), and T-cells (with substantial differences across multiple tissues, including 36.66 TPM in bone marrow and 44.3 TPM in PBMC). Additionally, macrophages show significant expression, especially in tissues such as the lymph node (24.8 TPM) and spleen (24.2 TPM). In contrast, non-immune tissues generally exhibit low to negligible expression levels of TSPAN32, with the exception of cardiomyocytes (14.5 TPM) (Figure 1A).

**Figure 2:**
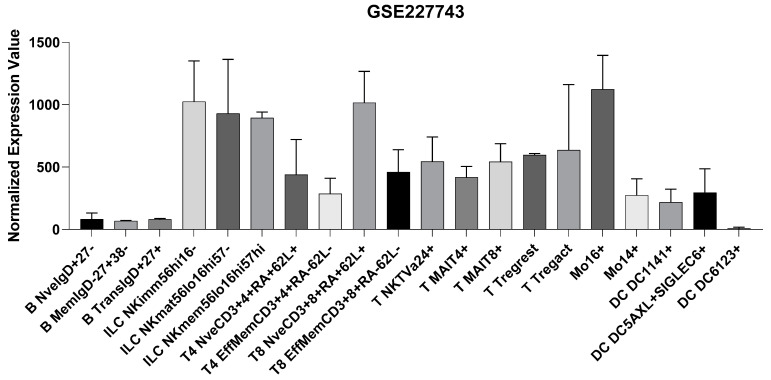
Expression levels of TSPAN32 in immune cell populations. Transcriptomic levels of TSPAN32 in different lymphoid and myeloid cells, as obtained from the GSE227743 dataset.

Based on the above data, we have interrogated the GSE227743, which included whole-genome transcriptomic analysis of various immune cells. NK cells, particularly, exhibit higher expression levels of TSPAN32 (Figure 2). Among T cells, naive CD8+ T cells (T8 NveCD3+8+RA+62L+) showed the highest expression of TSPAN32, followed by activated T regulatory cells (T Tregact) and NK T-cells (T NKTVa24+). Effector memory T-cells, both CD4+ (T4 EffMemCD3+4+RA-62L-) and CD8+ (T8 EffMemCD3), immature NK cells (ILC NKimm56hi16-, 1024.5 TPM), mature NK cells (ILC NKmat56lo16hi57-, 929.5 TPM), and memory NK cells (ILC NKmem56lo16hi57hi, 894.5 TPM) have moderate levels of TSPAN32 (Figure 2). Monocyte subsets show variable expression of TSPAN32, with Mo16+ cells exhibiting very high levels, compared to lower expression in Mo14+ cells. Also, dendritic cells vary greatly, with DC5AXL+SIGLEC6+ cells expressing more TSPAN32 than DC1141+ and DC6123+ subsets (Figure 2).

### 3.2. Transcription Factor Analysis

**Figure 3:**
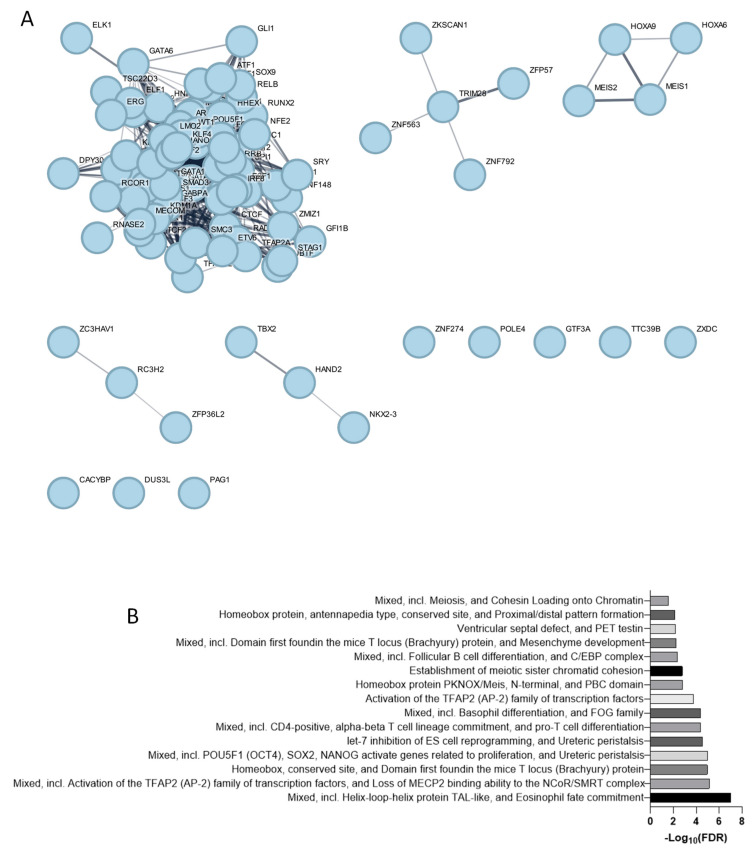
Transcription factors regulating TSPAN32. Network of TF involved in TSPAN32 regulation (**A**); StringDB clusters showing the functional role of TFs involved in TSPAN32 expression (**B**).

The analysis of the transcription factors controlling the expression of TSPAN32, as identified through publicly available ChIP-seq databases (Figure 3A), reveals a diverse array of functional roles (Figure 3B). One key group of transcription factors includes helix–loop–helix proteins like TAL1, which are involved in eosinophil fate commitment, crucial for hematopoiesis and lineage specification (FDR = 9.65 × 10^−8^). The TFAP2 (AP-2) family of transcription factors (FDR = 7.41 × 10^−6^) plays a significant role in gene activation and developmental processes, including the loss of MECP2 binding ability to the NCoR/SMRT complex, suggesting a regulatory influence on chromatin remodeling. Homeobox proteins, including those with domains first identified in the mice T locus (Brachyury) protein (FDR = 1.10 × 10^−5^), are essential for developmental processes and morphogenesis, regulating body structure formation during early development. Transcription factors such as POU5F1 (OCT4), SOX2, and NANOG (FDR = 1.10 × 10^−5^) are involved in the maintenance of pluripotency and reprogramming cells, activating genes related to proliferation and development, which is crucial in stem cell biology and differentiation. Additionally, factors involved in CD4-positive, alpha-beta T cell lineage commitment, and pro-T cell differentiation (FDR = 4.12 × 10^−5^) highlight their role in the development and function of immune cells. Lastly, transcription factors like RAD21 and SMC3, which are involved in establishing meiotic sister chromatid cohesion (FDR = 1.70 × 10^−3^), underscore the importance of accurate chromosome segregation during cell division (Figure 3B).

### 3.3. Gene Set Enrichment Analysis of Genes Correlated with TSPAN32 upon Immune Cell Activation

**Figure 4:**
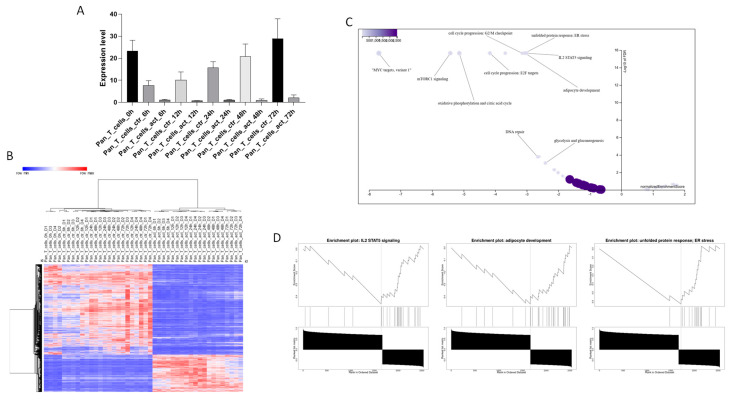
TSPAN32 in T cells activation. Modulation of TSPAN32 upon Pan-T cell activation, obtained from the GSE227743 dataset (**A**). Heatmap of genes significantly correlated with TSPAN32 upon Pan-T cell activation (**B**). Volcano plot of biological processes enriched by the genes significantly correlated with TSPAN32 (**C**). Top 3 biological processes enriched by the genes significantly correlated with TSPAN32, as obtained from the GSEA (**D**).

As shown in Figure 3A, TSPAN32 expression was significantly downregulated upon T cell activation. When analyzing the genes correlated with TSPAN32 upon T cell activation, we identified 3153 genes (FDR < 0.05 and correlation coefficient > |0.7|), out of which 2536 were unambiguously mapped to 2536 unique Entrez Gene IDs (Figure 4B). Among these 2536 unique Entrez Gene IDs, 629 IDs were annotated to the MSigDB Gene sets, which were used for the enrichment analysis. The analysis identified no positively related categories and 10 negatively related categories as enriched (Figure 4C,D).

The weighted set of enriched categories for redundancy reduction included the following significant Hallmark gene sets. The IL2 STAT5 signaling category had 28 genes, with 23 unique genes mapped, showing a correlation coefficient of −0.45994 and an NES of −3.0005 (FDR < 2.2 × 10^−16^) (Figure 4C,D). The adipogenesis category involved 30 genes and 24 unique mappings, with a correlation coefficient of −0.45664 and an NES of −3.0521 (FDR < 2.2 × 10^−16^) (Figure 4C,D). For the unfolded protein response, there were 17 genes, with 16 uniquely mapped, showing a correlation coefficient of −0.62443 and an NES of −3.0735 (FDR < 2.2 × 10^−16^) (Figure 4C,D). The G2M checkpoint category included 22 genes and 19 unique mappings, with a correlation coefficient of −0.55043 and an NES of −3.1448 (FDR < 2.2 × 10^−16^). The E2F targets comprised 35 genes with 31 unique mappings, a correlation coefficient of −0.57521, and an NES −4.1706 (FDR < 2.2 × 10^−16^). Oxidative phosphorylation and the citric acid cycle category included 54 genes and 50 unique mappings, with a correlation coefficient of −0.59662 and an NES −5.1402 (FDR < 2.2 × 10^−16^). The mTORC1 signaling category had 49 genes with 47 unique mappings, a correlation coefficient of −0.64253, and an NES of −5.4258 (FDR < 2.2 × 10^−16^). The MYC targets variant 1 category included 86 genes, all uniquely mapped, with a correlation coefficient of −0.68327 and an NES of −7.6892 (FDR < 2.2 × 10^−16^). The DNA repair category comprised 27 genes, with 16 unique mappings, showing a correlation coefficient of −0.42174 and an NES of −2.6538 (FDR = 0.00016929). Finally, the glycolysis and gluconeogenesis category included 31 genes with 22 unique mappings, a correlation coefficient of −0.35291, and an NES of −2.4114 (FDR = 0.00084644 (Figure 4C).

**Figure 5:**
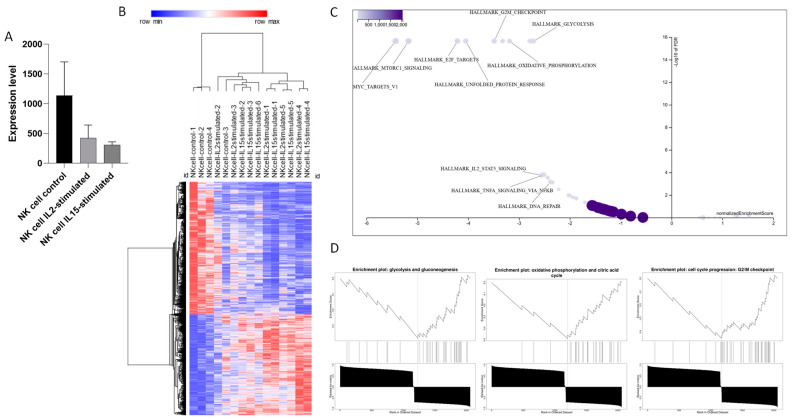
TSPAN32 in NK cells activation. Modulation of TSPAN32 upon Pan-T cell activation, obtained from the GSE197067 dataset (**A**). Heatmap of genes significantly correlated with TSPAN32 upon NK cell activation (**B**). Volcano plot of biological processes enriched by the genes significantly correlated with TSPAN32 (**C**). Top 3 biological processes enriched by the genes significantly correlated with TSPAN32, as obtained from the GSEA (**D**).

TSPAN32 underwent a significant downregulation in NK cells upon stimulation with both IL2 and IL15, as shown in Figure 5A. When analyzing the genes correlated with TSPAN32 upon NK cell activation, we identified 2323 genes (Figure 5B). Out of these, 2060 user IDs were unambiguously mapped to 2060 unique EntrezGene IDs, while 263 user IDs could not be mapped to any EntrezGene ID. Among the 2060 unique EntrezGene IDs, 613 IDs were annotated to the selected functional categories, which were used for the enrichment analysis. The analysis revealed no positively related categories but identified 10 negatively related categories as enriched (Figure 5C,D). The glycolysis and gluconeogenesis category involved 37 genes, with 29 in the leading edge, showing an NES of −2.7125 (FDR < 2.2 × 10^−16^). Oxidative phosphorylation and the citric acid cycle category comprised 30 genes, with 27 in the leading edge, exhibiting an NES of −3.1855 (FDR < 2.2 × 10^−16^). The G2M checkpoint comprised 42 genes, with 35 in the leading edge, exhibiting an NES of −3.4883 (FDR < 2.2 × 10^−16^). The unfolded protein response category involved 40 genes, with 34 in the leading edge, showing an NES of −4.0474 (FDR < 2.2 × 10^−16^). The E2F targets category included 52 genes, with 46 in the leading edge, showing an NES of −4.2119 (FDR < 2.2 × 10^−16^). The mTORC1 signaling category had 71 genes, with 59 in the leading edge, displaying an NES of −5.1721 (FDR < 2.2 × 10^−16^). The MYC targets variant 1 category included 81 genes, with 71 in the leading edge, showing an NES of −5.4265 (FDR < 2.2 × 10^−16^). The TNFA signaling via NF-κB category included 34 genes, with 26 in the leading edge, displaying an NES of −2.5128 (FDR = of 0.00015600). The IL2 STAT5 signaling category had 38 genes, with 30 in the leading edge, showing an NES of −2.5551 (FDR= 0.0001701). Lastly, the DNA repair category comprised 28 genes, with 21 in the leading edge, displaying an NES of −2.3826 (FDR= of 0.00066859) (Figure 5C,D).

### 3.4. Biological Processes Modulated upon TSPAN32 Genetic Ablation

To identify the biological processes modulated upon TSPAN32 genetic ablation, we utilized the SigCom consensus signature for TSPAN32_KO cells, available on Enrichr. We conducted an ORA separately for the downregulated and upregulated genes using Enrichr. The analysis included a list of 245 upregulated and 245 downregulated genes.

**Figure 6:**
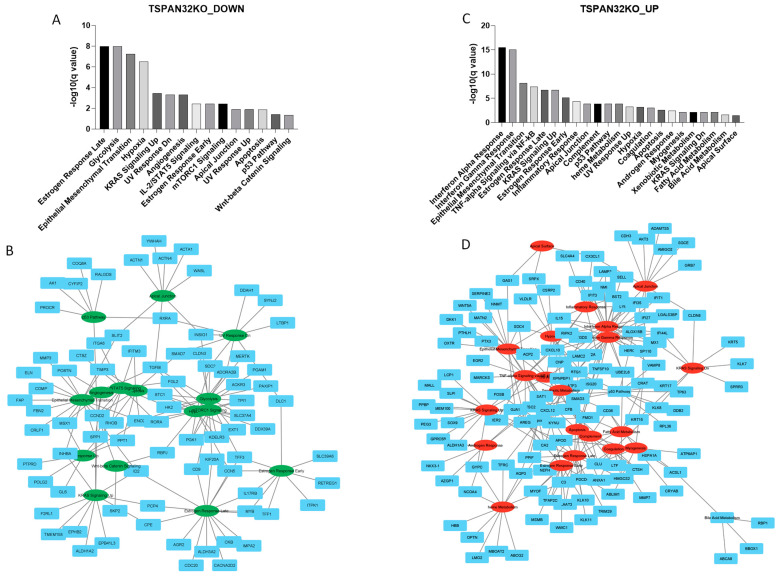
Biological processes regulated by TSPAN32. Biological processes enriched among the downregulated genes in the TSPAN32_KO consensus signature from the LINCS L1000 database (**A**); network representation of biological processes enriched among the downregulated genes in the TSPAN32_KO consensus signature (**B**); biological processes enriched among the upregulated genes in the TSPAN32_KO consensus signature from the LINCS L1000 database (**C**); network representation of biological processes enriched among the upregulated genes in the TSPAN32_KO consensus signature (**D**).

For the downregulated genes, the most significant enriched terms included Estrogen Response Late, glycolysis, Epithelial–Mesenchymal Transition, Hypoxia, and KRAS Signaling Up (Figure 6A,B). Estrogen Response Late showed 17 out of 200 genes overlapping, with an FDR of 1.04 × 10^−8^ and a combined score of 171.5869. The associated genes were ITPK1, CACNA2D2, IL17RB, CDC20, ALDH3A2, PCP4, IMPA2, ID2, MYB, CCN5, TFF3, AGR2, CPE, CD9, TFF1, KIF20A, and CKB. Glycolysis also had 17 out of 200 genes overlapping, including genes such as PAXIP1, TPI1, SDC2, PGAM1, STC1, ENO2, MERTK, HK2, EXT1, CLDN3, ADORA2B, TFF3, PGK1, KDELR3, KIF20A, TGFBI, and SLC37A4 (FDR = 1.04 × 10^−8^ and combined score = 171.5869). Epithelial–Mesenchymal Transition, with 16 out of 200 genes overlapping, had an FDR of 5.52 × 10^−8^ and a combined score of 144.4815, involving genes like FBN2, POSTN, ELN, MMP3, INHBA, ENO2, RHOB, COMP, FAP, ID2, SPP1, TIMP3, MSX1, TGFBI, SLIT2, and CRLF1. Hypoxia, showing 15 out of 200 genes overlapping, had an FDR of 3.08 × 10^−7^ and a combined score of 120.2851, with genes including TPI1, SDC2, STC1, RORA, ENO2, RBPJ, HK2, EXT1, ADORA2B, CCN5, PGK1, ACKR3, KDELR3, TGFBI, and SLC37A4. Lastly, KRAS Signaling Up had 11 out of 200 genes overlapping, with an FDR of 0.000348 and a combined score of 49.5513, including genes such as PCP4, TMEM158, CCND2, ID2, ALDH1A2, EPB41L3, SPP1, F2RL1, CPE, INHBA, and EPHB2 (Figure 6A,B).

For the upregulated genes, the most significant enriched terms were Interferon Alpha Response, Interferon Gamma Response, Epithelial–Mesenchymal Transition, TNF-alpha Signaling via NF-kB, and Estrogen Response Late.

Interferon Alpha Response had 19 out of 97 genes overlapping, with an FDR of 3.15 × 10^−16^ and a combined score of 837.7665, involving genes such as LGALS3BP, CNP, SP110, IL15, RIPK2, MX1, UBE2L6, IFI35, NMI, IFIT3, IFI44L, BST2, ISG20, CXCL10, IFI27, SELL, LAMP3, HERC6, and LY6E. Interferon Gamma Response, with 24 out of 200 genes overlapping, had an FDR of 9.05 × 10^−16^ and a combined score of 456.0704, including genes like LGALS3BP, CD40, BTG1, SP110, IL15, RIPK2, MX1, TNFAIP3, UBE2L6, IFI35, NMI, IFIT1, IFIT3, IFI44L, BST2, VAMP8, ISG20, CXCL10, MT2A, IFI27, TNFSF10, CFB, HERC6, and LY6E. Epithelial–Mesenchymal Transition had 17 out of 200 genes overlapping, with an FDR of 6.78 × 10^−9^ and a combined score of 171.5869, involving genes such as OXTR, NNMT, SERPINE2, SDC4, IL15, WNT5A, TNFAIP3, LAMC2, DKK1, AREG, SAT1, PTHLH, GJA1, CXCL12, GAS1, PTX3, and MATN2. TNF-alpha Signaling via NF-kB, with 16 out of 200 genes overlapping, had an FDR of 4.05 × 10^−8^ and a combined score of 144.4815, including genes like EGR2, BTG1, SMAD3, SDC4, TSC22D1, RIPK2, TNFAIP3, AREG, SAT1, CXCL10, MARCKS, KYNU, FOSB, PTX3, IER2, and ATF3. Lastly, Estrogen Response Late had 15 out of 200 genes overlapping, with an FDR of 2.01 × 10^−7^ and a combined score of 120.2851, involving genes such as TFAP2C, MYOF, PLAAT3, LAMC2, AREG, KLK10, KLK11, ISG20, CXCL12, TRIM29, CA2, PPIF, PDCD4, HMGCS2, and LTF (Figure 6C,D).

## 4. Discussion

Our study confirms and expands upon the known high expression of TSPAN32 in immune cells, including NK cells, monocytes, and various T-cell subsets. This robust expression profile strongly suggests a significant role for TSPAN32 in immune cell development, activation, and function. The transcription factor analysis further elucidates the regulatory networks controlling TSPAN32 expression, highlighting the involvement of TAL1 and TFAP2 family members, among others, which are well-established contributors to hematopoiesis and immune cell lineage commitment, indicating that TSPAN32 regulation is finely tuned to influence immune cell maturation and differentiation. Additionally, the potential roles of pluripotency-associated transcription factors such as POU5F1, SOX2, and NANOG in controlling TSPAN32 suggest its involvement in broader developmental and reprogramming contexts, including possible implications for onco-hematological disorders, such as chronic myeloid leukemia [17].

Moreover, our gene set enrichment analyses revealed important insights into TSPAN32 function. The observed downregulation of TSPAN32 during immune cell activation suggests that it takes part as a negative regulator of immune responses, participating with other factors in modulating critical metabolic and signaling pathways. This is in line with previous studies, showing a role for TSPAN32 in autoimmune disorders [7,8,9,18,19]. In addition, the characterization of the enriched biological processes upon TSPAN32 knockout highlights its impact on cellular physiology and immune responses. The upregulation of genes associated with responses to interferons and TNF-alpha Signaling pathways suggests that TSPAN32 plays an active role in the regulation of cellular differentiation and immune cell activation. These findings highlight the complex regulatory network in which TSPAN32 participates, influencing diverse biological processes critical for cellular homeostasis and immune function. Nevertheless, while these findings suggest regulatory roles, we clearly acknowledge that these relationships are correlative and provide the background for further experimental validation to confirm causative mechanisms.

Existing evidence suggests that TSPAN32 expression remains modulated in memory T cells. As reported by Basile et al., 2020 [7] memory CD4+ T effector cells exhibit significantly lower TSPAN32 expression levels compared to naïve T cells. Additionally, restimulation of memory T cells was associated with further downregulation of TSPAN32 expression. Notably, this modulation was specific to effector T cells, as no changes were observed in memory Treg cells. These findings suggest that the regulation of TSPAN32 may be a sustained feature in differentiated or activated T cell subsets.

The observation of the activation of the type I interferon response upon TSPAN32_KO is noteworthy, which is in accordance with our previous observation in SLE [9]. Indeed, our findings indicate that TSPAN32 is significantly downregulated in SLE plasmablasts, and its expression inversely correlates with genes involved in various immune-related pathways, including antigen processing and presentation, cell cycle regulation, and type I interferon signaling [9]. The involvement of IFN-alpha in SLE is well-documented [20,21]. Genome-wide association studies (GWASs) have identified over 100 susceptibility loci for SLE, many of which are involved in the type I IFN pathway. Specifically, polymorphisms in genes like IRF5, IRF7, STAT4, IFIH1, and UBE2L3, which have increased functional activity, are associated with SLE. This association results in elevated levels of circulating IFN-α or increased sensitivity to IFN-α in SLE patients. Correspondingly, IFN-α significantly reduces TSPAN32 levels in B cells in vitro, and transgenic mice overexpressing IFN-α (IFN-α Tg mice) show markedly decreased TSPAN32 levels in their splenocytes. IFN-α is a well-known critical factor in SLE development, and its overexpression in mice leads to symptoms similar to SLE, such as serum immune complexes, anti-dsDNA antibodies, glomerulonephritis, and hair loss. Consistent with these findings, genetic inhibition of IFN-alpha signaling protects mice from SLE [22], and the anti-IFN-α monoclonal antibody anifrolumab has been recently approved by the FDA for treating SLE patients [23,24,25].

While our data provide intriguing evidence of a relationship between TSPAN32 expression and type I interferon signaling, the mechanistic role of TSPAN32 in this process remains to be fully elucidated. Indeed, while interferons downregulate TSPAN32 expression, as we have previously shown in vitro and in IFN-α transgenic mice [9], on the other hand, TSPAN32 knockout is associated with the enrichment of type I interferon signaling pathways. This dual relationship could reflect a regulatory feedback loop, where TSPAN32 serves as a negative regulator of interferon signaling, and its downregulation or absence may lead to compensatory activation of these pathways. One possible explanation is that TSPAN32 might function as a molecular brake on type I interferon signaling under normal conditions. Its downregulation by IFN-α could represent a mechanism to amplify interferon responses during an immune challenge. However, the complete absence of TSPAN32, as observed in knockout models, could result in dysregulated feedback, leading to overactivation of interferon-related pathways. This hypothesis aligns with observations in autoimmune diseases such as SLE, where type I interferon overexpression plays a central role in pathogenesis. The suppression of TSPAN32 by IFN-α might further exacerbate this cycle, creating a pro-inflammatory environment characterized by sustained interferon activity. The knockout data provide evidence for the involvement of TSPAN32 in this regulatory axis and suggest that loss of its function could promote immune dysregulation.

While our study focuses on the regulation of TSPAN32 under specific conditions, the broader implications of transient versus sustained modulation are critical to understanding its biological significance. Transient loss of TSPAN32 (for instance upon pharmacological intervention) may represent a reversible mechanism during T cell activation, potentially fine-tuning effector functions without permanently altering T cell identity. Conversely, definitive downregulation, as observed in memory CD4+ T effector cells (Basile et al., 2020 [7]), likely reflects stable epigenetic or transcriptional reprogramming, which could influence long-term immune responses and immune system homeostasis.

Furthermore, T cells appear to respond differently to TSPAN32 modulation compared to other immune cells. Tarrant et al., 2002 [5] showed that TSPAN32 gt/gt mice exhibit hyperactivation of T cells but not B cells, suggesting cell-specific roles for TSPAN32 in immune regulation. In addition, genetic studies provide compelling evidence linking TSPAN32 to autoimmune diseases. Genome-wide association studies (GWASs) have implicated TSPAN32 SNPs in autoimmunity. Hong et al. identified SNP rs2074023 within TSPAN32 associated with inflammatory bowel disease (IBD) [26]. This SNP, located in a linkage disequilibrium region with several genes, is inversely associated with TSPAN32 expression in lymphoblastoid cell lines and the small intestine. Another GWAS meta-analysis on systemic sclerosis (SSc) found SNP rs2651804 near TSPAN32 strongly associated with SSc, particularly the limited cutaneous subtype. This SNP also influences gene expression in whole blood, indicating its role in SSc pathophysiology and the complex regulatory mechanisms underlying the disease [27].

These findings collectively suggest that transient versus definitive modulation of TSPAN32 may play a pivotal role in shaping immune responses and disease susceptibility. Future studies should investigate the context-dependent nature of these regulatory mechanisms and their differential effects on immune cell subsets, with potential implications for autoimmunity, chronic inflammation, and immune-related malignancies.

Furthermore, GWASs have identified several significant associations between single nucleotide polymorphisms (SNPs) and various hematological and immunological traits. For example, the missense variant rs61744929 has been linked to multiple blood cell parameters. This SNP shows a strong association with increased hemoglobin levels and higher mean corpuscular hemoglobin concentration. Furthermore, rs61744929 correlates with a decrease in neutrophil count and an increase in the lymphocyte percentage of white cells. Another SNP, rs58833930, an intron variant, is significantly associated with eosinophil counts, highlighting its potential impact on immune cell regulation. These findings underscore the critical role of specific genetic variations in influencing blood cell traits and immune responses, providing valuable insights for potential therapeutic targets.

The implications of our findings extend beyond basic biology to potential clinical applications. Understanding the role of TSPAN32 in immune cell regulation could lead to novel therapeutic strategies for immune-related disorders, including autoimmune diseases, cancer immunotherapy, and infectious diseases. Targeting TSPAN32 or its associated pathways may offer new avenues for modulating immune responses and enhancing immune surveillance against pathogens or cancer cells. However, up to now, no drug targeting tetraspanins, including TSPAN32, is currently approved for clinical use. However, therapeutic strategies such as monoclonal antibodies, recombinant soluble large extracellular loops, and RNA interference are being explored. An example is Otlertuzumab, targeting CD37, undergoing trials for certain lymphomas [28,29,30]. These advancements highlight the potential for tetraspanin-targeted therapies, paving the way for similar approaches using TSPAN32 in autoimmune diseases.

Recognizing the limitations of our study, we emphasize the need for experimental validation to confirm and expand upon our findings. While transcriptomic data provide a robust basis for hypothesis generation, functional studies are necessary to delineate the precise roles of TSPAN32 in immune processes and its broader biological implications. Also, it is important to note that direct information on the functional roles of TSPAN32 at the protein level is currently lacking. Insights into its potential functions can only be inferred based on the well-documented roles of other tetraspanin family members. Tetraspanins are known to organize specialized membrane microdomains, referred to as tetraspanin-enriched microdomains (TEMs), which facilitate the assembly of signaling complexes at the cell surface. These microdomains play a crucial role in regulating receptor–ligand interactions and downstream signaling pathways during immune cell activation. It is reasonable to hypothesize that TSPAN32 might similarly contribute to the spatial organization and modulation of immune signaling, helping to fine-tune receptor activation and maintaining cellular homeostasis.

In conclusion, our study presents a comprehensive analysis of TSPAN32, revealing its multifaceted role in immune regulation, cellular metabolism, and disease contexts. By integrating gene expression patterns, regulatory networks, and functional associations, we establish a framework for future research to explore the therapeutic potential of targeting TSPAN32 in immune-related and hematological disorders.

## Data Availability

All data used in this study are available at the sources detailed in the text.
